# Development of a multiplex methylation-specific PCR as candidate triage test for women with an HPV-positive cervical scrape

**DOI:** 10.1186/1471-2407-12-551

**Published:** 2012-11-23

**Authors:** Suzanne Snellenberg, Lise MA De Strooper, Albertus T Hesselink, Chris JLM Meijer, Peter JF Snijders, Daniëlle AM Heideman, Renske DM Steenbergen

**Affiliations:** 1 Department of Pathology, VU University Medical Center, Amsterdam, The Netherlands

**Keywords:** Cervical cancer, HPV testing, DNA methylation, Promoter, CADM1, MAL, hsa-miR-124-2, Multiplex qMSP, Primer/probe design, 7500Fast ABI system

## Abstract

**Background:**

Quantitative methylation-specific PCR (qMSP) analysis for determining the methylation status of (candidate) tumor suppressor genes has potential as objective and valuable test to triage high-risk human papillomavirus (hrHPV) positive women in cervical screening. Particularly combined methylation analysis of a panel of genes shows most promising clinical performance, with sensitivity levels that equal or exceed that of cytology. However, the wide application of such methylation marker panels is hampered by the lack of effective multiplex assays allowing simultaneous methylation detection of various targets in a single reaction. Here, we designed and analyzed a multiplex qMSP assay for three genes whose methylation was previously found to be informative for cervical (pre)cancer (i.e. CADM1, MAL and hsa-miR-124-2) as well as a reference gene β-actin. Based on our experience, we discuss the optimization of the parameters that provide a practical approach towards multiplex qMSP design.

**Methods:**

Primers and PCR reagents were optimized for multiplex qMSP purposes and the resulting assay was analytically validated on serial dilutions of methylated DNA in unmethylated DNA, and compared with singleplex counterparts on hrHPV-positive cervical scrapings.

**Results:**

Upon optimization, including primer redesign and primer limiting assays, the multiplex qMSP showed the same analytical performance as the singleplex qMSPs. A strong correlation between the obtained normalized ratios of the singleplex and multiplex qMSPs on cervical scrapes was found for all three markers: CADM1 (R^2^=0.985), MAL (R^2^=0.986) and hsa-miR-124-2 (R^2^=0.944).

**Conclusion:**

Multiplex qMSP offers a promising approach for high-throughput diagnostic analysis of the methylation status of multiple genes, which after proper design and validation can be equally specific, sensitive and reproducible as its singleplex versions.

## Background

Persistent infection with high-risk human papillomavirus (hrHPV) types is causally involved in the development of both squamous cell carcinoma (SCC) and adenocarcinoma (AdCA) of the cervix [1,2]. Testing for hrHPV in cervical screening programs results in earlier detection of clinically relevant cervical lesions (high grade cervical intraepithelial neoplasia or cancer (CIN2+) than cytology [3,4]. This provides a higher reassurance of low cervical cancer risk in test negative women [4,5]. However, only a fraction of hrHPV positive women will have or develop CIN2+, arguing for the use of additive disease markers to distinguish the subgroup of women having a high likelihood of high-grade disease in need of further gynecologic examination.

Epigenetic silencing of tumor suppressor genes by DNA methylation in cervical (pre)cancers has been shown to provide disease biomarkers with great potential applicable to both clinician-collected cervical scrape samples and self-collected cervico-vaginal specimens [6-9]. Methylation of CpG islands within promoter regions of genes and microRNAs such as CADM1, MAL, and hsa-miR-124-2, reflects mechanistically relevant events for cervical carcinogenesis [8,10,11]. Until now, DNA methylation of many genes has been analyzed, often by quantitative methylation-specific PCR (qMSP), on tissue and/or cervical scrape samples (reviewed by Wentzensen et al. [12]). Recent studies indicate that most optimal sensitivity rates for CIN2+ can only be obtained by testing for a combination of methylation markers [13-16]. However, determining the methylation status of multiple methylation markers separately is time consuming and relatively large amounts of sample material are needed. Multiplexing allows for more methylation targets to be analyzed using a single aliquot of sample material with potential for reducing target-to-target differences and monitoring sample adequacy for PCR purpose by an internal reference gene, thereby saving material, time and costs and improving quality control.

Here, we describe the consecutive experimental steps to set up a multiplex qMSP for CADM1, MAL and hsa-miR-124-2 and the reference gene β-actin (ACTB) with equal analytical performance as the individual singleplex qMSP assays. Following analytical validation, a proof of concept analysis was performed on cervical scrapings. The findings provide a practical guide for qMSP design and demonstrate that multiplex qMSP can be used for high-throughput diagnostic analysis, without the risk of a decrease in assay performance.

## Methods

### Cell cultures

Primary human foreskin keratinocytes (EKs) and the cervical cancer cell line SiHa were cultured as described previously [17].

### Cervical scrapings

Cervical scrapings were obtained from the population-based cervical screening trial POBASCAM, registered as ISRCTN20781131 [18]. We randomly selected 33 cervical scrapings of GP5+/6+−PCR hrHPV-positive women with normal cytology without evidence of CIN2+ up to the next screening round after 5 years (i.e., two had histologically CIN1, 31 had histologically no CIN) and 12 scrapings classified as mild dyskaryosis or worse of hrHPV-positive women with CIN3 (n=11) or SCC (n=1) diagnosed within 18 months of follow-up. This study followed the ethical guidelines of the Institutional Review Board of the VU University Medical Center.

### DNA extraction, HPV typing and bisulfite modification

DNA was isolated from cervical scrapes using Nucleo-Spin 96 Tissue kit (Macherey-Nagel) and a Microlab Star robotic system (Hamilton) according to manufacturers' instructions. Genomic DNA from cell cultures was isolated with UltraPure™ Phenol:Chloroform:Isoamyl Alcohol (Invitrogen Life Science Ltd, Carlsbad, CA USA). HPV detection and genotyping was performed using the general primer GP5+/6+−PCR enzyme immunoassay, followed by reverse line blot analysis [19]. Furthermore, genomic DNA from tissue specimens and cell lines (0.5 to 2 μg) were subjected to bisulfite treatment with the EZ DNA Methylation Kit™ (Zymo Research, Orange, CA, USA). Standard curves were generated by spiking methylated DNA from the SiHa cell line in unmethylated DNA from EKs in order to obtain a serial dilution of 50 ng to 0.25 ng methylated DNA in a total of 50 ng of DNA.

### Quantitative MSP (qMSP) analysis

For the amplification reaction 2.5 μl bisulfite treated DNA (50 ng) was added to 10 μl amplification mix containing 1x Quantitect Probe mix (Qiagen, Leusden, The Netherlands), various primer concentrations (50 – 400 nM) and 200 nM of the hydrolysis probe. Sequences of primers and hydrolysis probes are available on request. Amplification and real-time measurement was performed in the 7500Fast ABI system (Applied Biosystems, Foster City, CA, USA), using the following conditions; 15 min at 95°C followed by 40 cycles of 15 s at 95°C and 1 min at 60°C.

### Multiplex qMSP analysis

Multiplex qMSP analysis was mainly done using QuantiTect Muliplex mix (Qiagen), unless specified otherwise. The other master mixes tested were EpiTect MethyLight mix (Qiagen), iQ Multiplex Powermix (Bio-Rad, Veenendaal, The Netherlands) or Genotyping Master Mix (Applied Biosystems). For the amplification reaction, 2.5 μl bisulfite treated DNA (50 ng) was added to 10 μl amplification mix containing 1x Multiplex mix, 400 nM of MAL and hsa-miR-124-2 primer, 200 nM of ACTB and CADM1 primer, and 200 nM hydrolysis probe of each target with the following conditions; 10 or 15 min at 95°C followed by 40 cycles of 15 s or 60 s at 94°C or 95°C and 60 s or 90 s at 60°C, depending on the buffer system used.

### Data analysis

Methylation values were normalized to the reference gene ACTB using the comparative Cq method (2^-ΔCQ^) [20]. The amplification efficiencies were calculated by E=(10^(−1/Slope)^-1)×100% for all serial dilutions [21]. Correlations (R^2^) of the serial dilutions and normalized ratios between the multiplex and singleplex qMSP of cervical scrapings were determined using Microsoft® Office Excel 2003 (SP3).

## Results

### Parameters important for multiplex qMSP design

**Figure 1:**
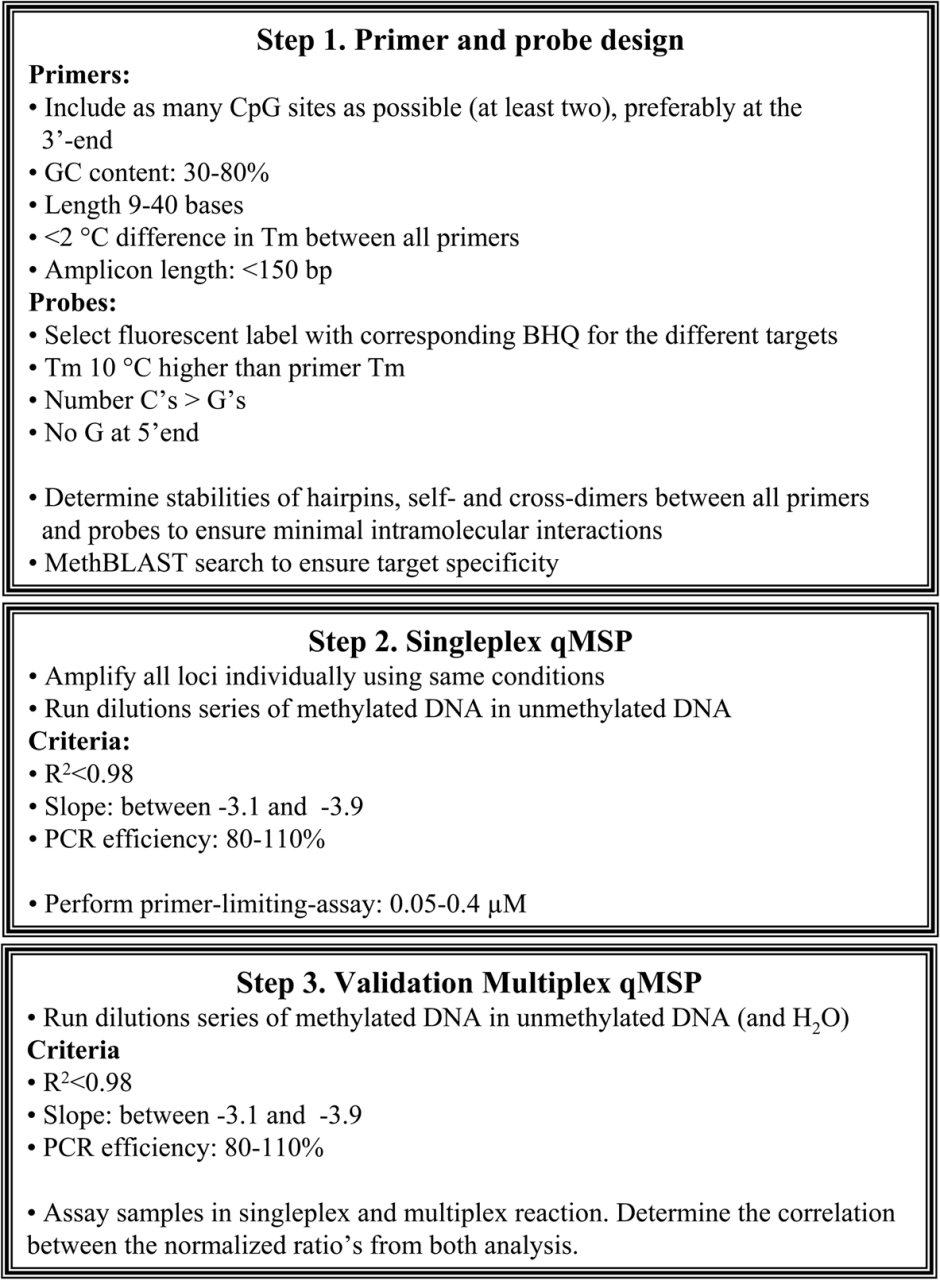
**Practical approach to multiplex qMSP development.** Partially adapted from Henegariu [22].

The various parameters important for multiplex qMSP development, as summarized in Figure 1, are described in the following section.

First, multiplex qMSP development requires the selection of dyes that give a good spectral separation to avoid overlap of the signals of different targets. The ABI7500Fast Real-Time PCR System, used in this study, has five different channels. One channel is used by ROX (emission maximum at 602 nm), a dye present in the master mix to correct for pipetting errors (passive reference), leaving four channels to be used for target detection. In this study, FAM (520 nm), JOE (548 nm), Dragon Fly Orange (DFO; 576 nm) and CY5 (650 nm) were used. It should be taken into account that the fluorescence intensities (ΔRn) differ between these four dyes, which can affect the Quantification Cycle (Cq) values.

**Figure 2:**
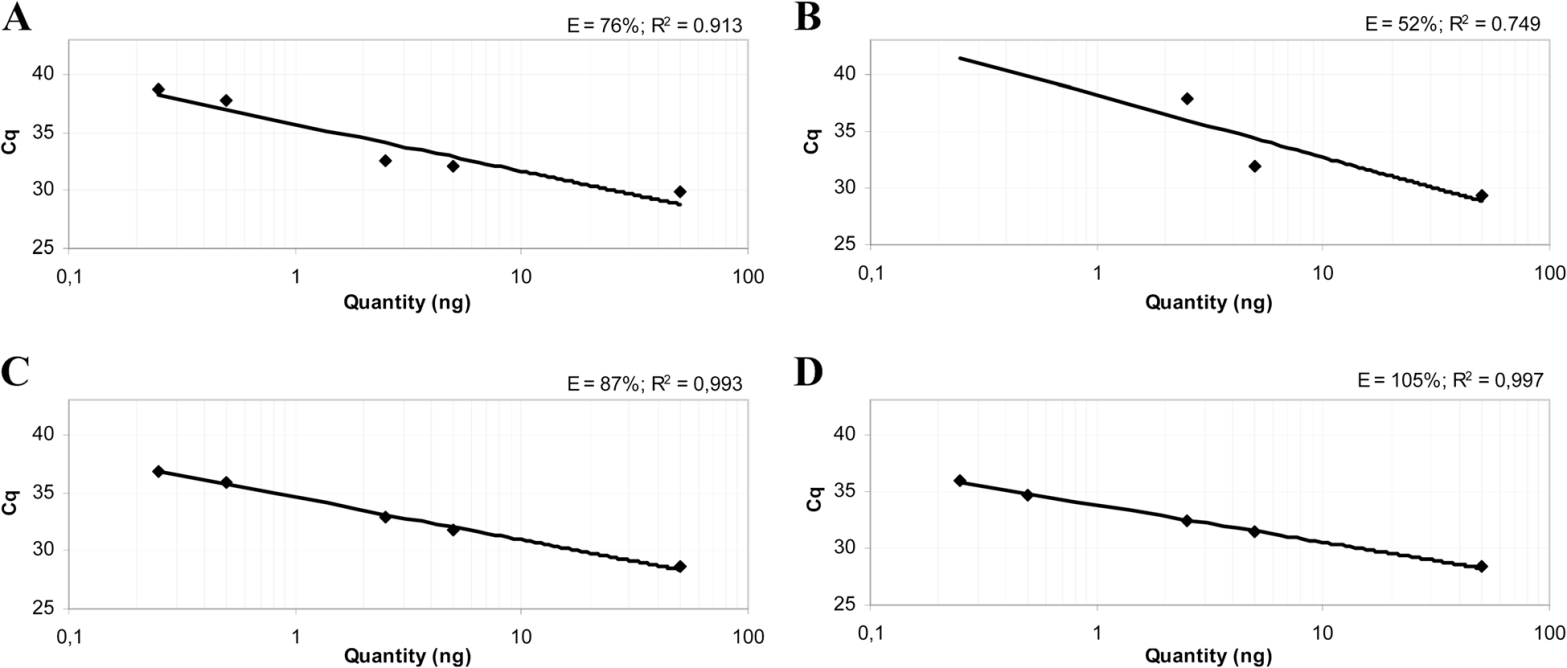
Standard curves of CADM1 and MAL in a multiplex qMSP with ACTB^* ^**tested on a serial dilution series of methylation positive SiHa DNA in methylation negative DNA of primary keratinocytes.** Standard curve of CADM1 (**A**) and MAL (**B**) with the previously described primers ^9^. CADM1 (**C**) and MAL (**D**) primers with nearly identical annealing temperatures as ACTB primers. For the dilution series the PCR efficiencies were calculated by E=(10^(−1/Slope)^-1)×100%. The qPCR efficiencies increased from 76% to 87% for CADM1 and 52% to 105% for MAL.^*^) Multiplex qMSP was performed with an ACTB primer concentration of 200 nM.

The second step involves the selection of primer pairs for all targets that display nearly identical annealing temperatures in order to ensure similar amplification efficiencies. Singleplex qMSPs for CADM1, MAL and hsa-miR-124-2 have been described previously [9,11], though primers differed in their annealing temperatures as determined using Primer Express version 3.0 (Applied Biosystems). Whereas the annealing temperatures of the hsa-miR-124-2 primers and primers of ACTB were comparable (58.2-59.9°C), the CADM1 and MAL primers had lower annealing temperatures (54-57°C). When testing these primer pairs in a multiplex qMSP at an annealing temperature of 60°C, no linear amplification curves were seen for CADM1 and MAL (Figure 2A, B). Therefore, CADM1 and MAL primers were redesigned to obtain primers with annealing temperatures comparable to that of ACTB and hsa-miR-124-2. For primer (re)design the following parameters were taken into account: 1) inclusion of as many CpG sites as possible (at least two), preferably at the 3’-end [23]; 2) maximum amplicon length of 150 bp, based on the fact that DNA is strongly degraded upon bisulfite conversion; 3) minimization of formation of intramolecular interactions within the primer (hairpins), dimerization with itself (self-dimers) or with primers for the other targets (cross-dimers) and 4) a methBLAST search at http://medgen.ugent.be/methBLAST/ to ensure target specificity of primers.

Following primer redesign, the PCR efficiencies increased from 75% to 95% and 52% to 105% for CADM1 and MAL, respectively, in the multiplex qMSP (Figure 2C, D).

**Figure 3:**
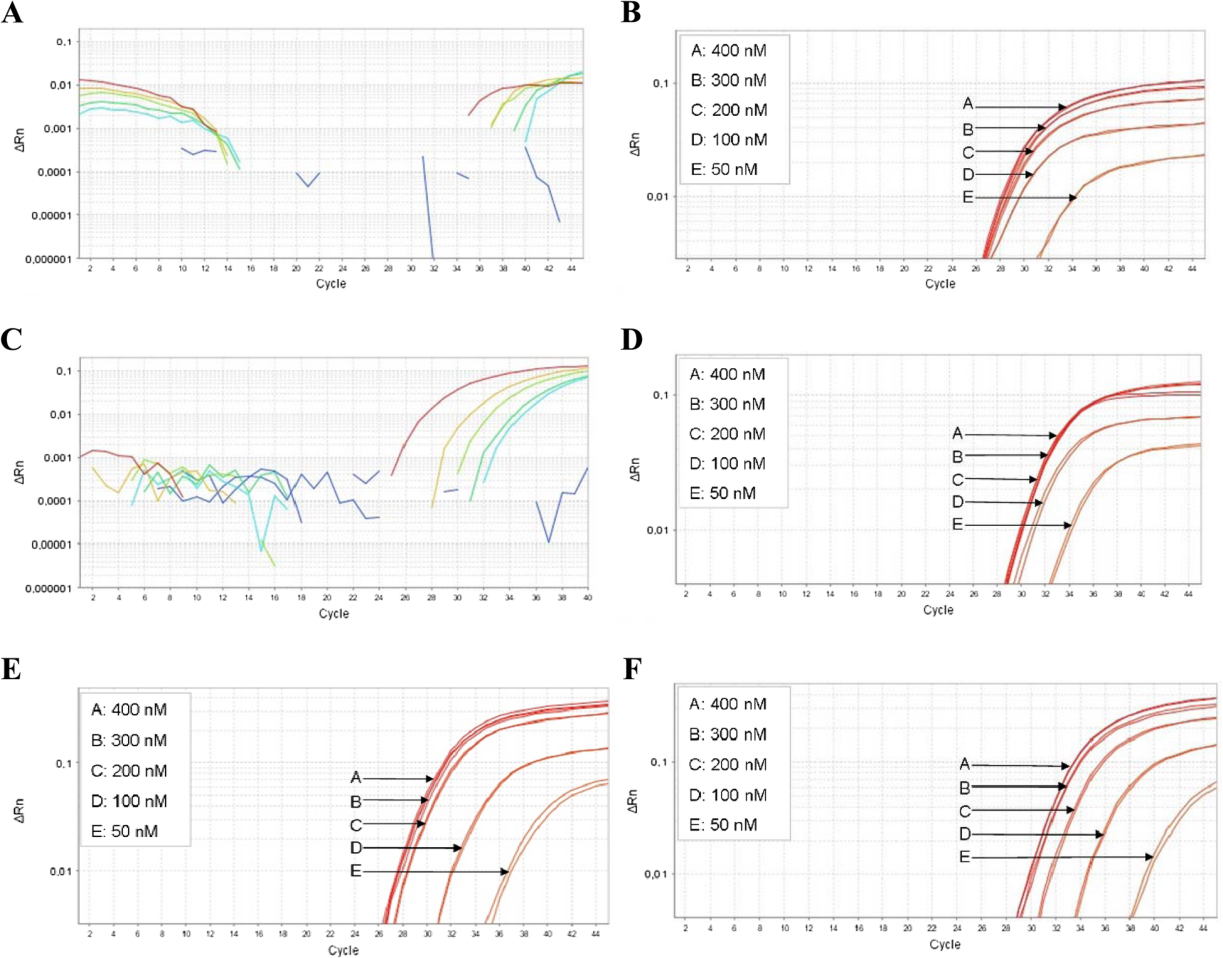
**Primer-limiting assays for ACTB, CADM1, MAL and hsa-miR-124-2.** (**A**) Amplification curve of MAL in a multiplex qMSP with ACTB. When ACTB-primers are used at a concentration of 400 nM, there is little to no amplification of MAL, which could be due to dNTP depletion by ACTB. (**B**) The minimum primer concentration of ACTB was 200 nM. (**C**) When the ACTB-primer concentration was lowered to 200 nM MAL amplification was not hampered. The minimum primer concentration was 200 nM for CADM1 (**D**), and 400 nM for MAL (**E**) and miR-124-2 (**F**).

Next a primer limiting assay was performed to determine at which primer concentration an early plateau phase is reached, resulting in a lower ΔRn, with an unchanged Cq. This optimization is required because amplification of most abundant targets may result in depletion of the dNTPs present in the reaction mixture, thereby hampering amplification of other targets (Figure 3A). To determine the lowest primer concentration that does not affect the Cq value for all four targets, singleplex qMSP was performed with different primer concentrations, ranging from 400 nM to 50 nM. For ACTB the same Cq was obtained at primer concentrations of 400, 300 and 200 nM, with a lower ΔRn observed at 300 and 200 nM (Figure 3B). The Cq increased at a primer concentration of 100 nM. Hence, 200 nM was selected as the minimum concentration for amplifying ACTB in a multiplex reaction. When an ACTB-MAL multiplex qMSP was performed with an ACTB-primer concentration of 200 nM, MAL is efficiently amplified (Figure 3C). For CADM1, MAL and hsa-miR-124-2 the minimum primer concentrations were 200 nM, 400 nM and 400 nM (Figure 3D-F), respectively.

To optimize the multiplex qMSP analysis, various commercially available TaqMan buffer systems were compared. For this purpose, the EpiTect MethyLight Mix (Qiagen), the iQ Multiplex Powermix (Bio-Rad), the Genotyping Master Mix (Applied Biosystems) and the QuantiTect Multiplex Mix (Qiagen) were tested on 50 ng of bisulfite treated SiHa DNA, which is positive for CADM1, MAL and hsa-miR-124-2 methylation. The reactions were performed in triplicate using multiplex qMSP conditions as outlined in the Materials and Methods section. The fluorescence data with the different TaqMan buffer systems were collected to determine the Cq at a threshold of 0.01 and the highest maximum fluorescent signal (ΔRn at Cq=40). The most optimal TaqMan buffer system for multiplex qMSP should have a low Cq value combined with a high ΔRn. The Genotyping Master Mix gave the highest Cq values combined with the highest ΔRn values, indicating that this master mix is not ideal for multiplex qMSP (Table 1). Reactions that used iQ Multiplex Powermix revealed the highest maximum ΔRn. However, as the minimum ΔRn (Cq=0) was relatively high, the threshold needed to be adjusted to 0.1. This adjustment resulted in the lowest Cq values, which could not be compared to the Cq values obtained with the other reaction mixtures due to differences in thresholds. With the iQ Multiplex Powermix the amplification curves for all targets had relatively small exponential phases, resulting in a small range for determining the most optimal threshold. The Cq and ΔRn values obtained with EpiTect Methylight and QuantiTect Multiplex buffer systems were comparable. Due to the convenience of the presence of the passive reference ROX in the QuantiTect Multiplex buffer, which was not in the EpiTect Methylight mix for multiplexing on a ABI7500, the QuantiTect Multiplex mix was used in subsequent optimization experiments.

**Table 1:** Multiplex qMSP results using different TaqMan buffer systems^*^

**TaqMan Buffer System**					
**Primers**	**Parameter**	**EpiTect MethyLight Mix (Qiagen)**	iQ Multiplex Powermix (Bio-Rad)^**^	**Genotyping Master Mix (ABI)**	**QuantiTect Multiplex Mix (Qiagen)**
**ACTB**	**Cq**	27.19±0.093	26.712±0.072	28.448±0.933	27.972±0.032
	**ΔRn**	0.244±0.004	2.655±0.003	0.791±0.090	0.170±0.003
**CADM1**	**Cq**	28.216±0.130	27.485±0.038	28.463±0.341	28.888±0.103
	**ΔRn**	0.267±0.006	2.722±0.038	1.160±0.056	0.182±0.004
**MAL**	**Cq**	26.432±0.170	25.593±0.030	29.499±0.837	26.759±0.053
	**ΔRn**	0.371±0.022	2.662±0.026	1.003±0.203	0.282±0.023
**Hsa-miR-124-2**	**Cq**	27.062±0.164	25.861±0.144	29.330±0.826	27.545±0.021
	**ΔRn**	0.630±0.010	6.505±0.029	0.691±0.125	0.514±0.014

^*^ Triplicate analyses of 50 ng of bisulfite treated SiHa DNA. Mean values are indicated ± standard deviations.

^**^ Threshold 0.1.

### Analytical validation of the multiplex qMSP

**Figure 4:**
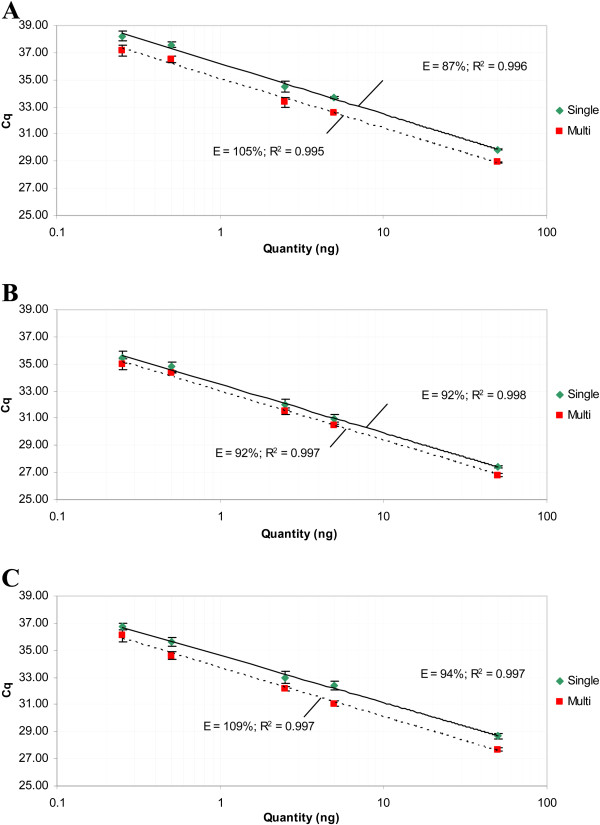
**High linearity between singleplex and multiplex analysis on serial dilutions of methylated DNA (SiHa) in unmethylated DNA (EK).** The multiplex reactions showed comparable efficiencies as the singleplex reactions for CADM1 (**A**), MAL (**B**) and hsa-miR-124-2 (**C**). Same thresholds were used in singleplex and multiplex qMSP, resulting in different Cq values for all targets. When adjusting the thresholds, the same Cq values can be obtained, resulting in overlap of the standard curves.

Using a serial dilution series of methylated DNA in unmethylated DNA, the performance of the designed multiplex qMSP was compared to the singleplex qMSP (Figure 4). The correlation (R^2^) for all the singleplex and multiplex qMSPs were higher than 0.99, indicating high linearity (Figure 4A-C). Whereas the Cq values, when determined at a threshold of 0.01, differed between singleplex and multiplex qMSP, same Cq values were obtained when adjusting the thresholds, resulting in overlapping standard curves. In addition, high efficiencies, ranging from 87% to 94% for singleplex qMSP and 92% to 109% for multiplex qMSP, were found. This indicates that there is no qMSP inhibition upon multiplexing ACTB, CADM1, MAL and hsa-miR-124-2. The high reproducibility of the multiplex qMSP is demonstrated in Additional file 1, showing the results of 10 independent multiplex qMSP runs on a serial dilution of 50 to 0.05 ng of methylated DNA in a background of unmethylated DNA.

### Evaluation of multiplex qMSP on cervical scrapings

**Figure 5:**
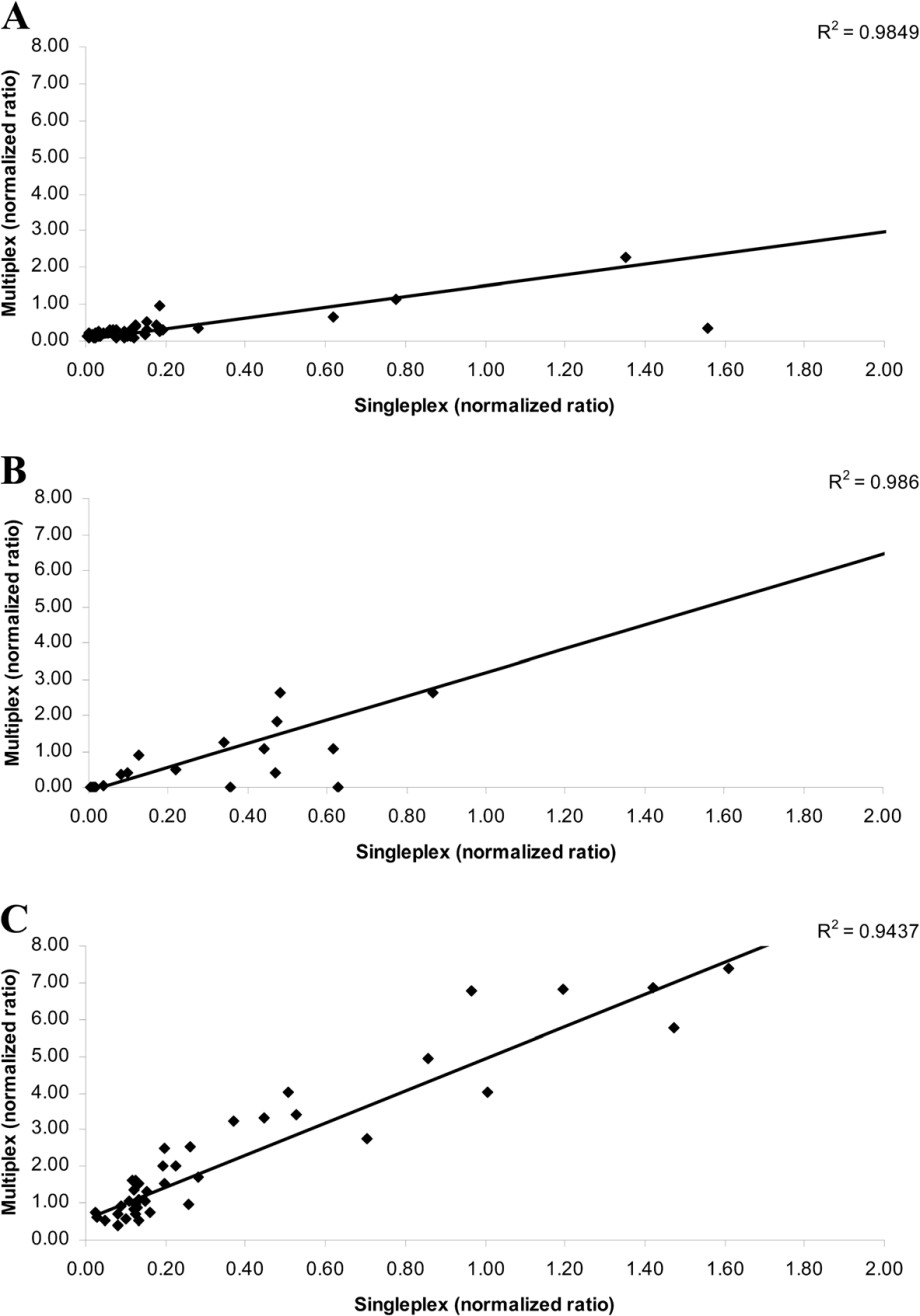
**Correlation between the ΔCq values obtained with singleplex and multiplex qMSP.** The R^2^ values for the three markers were 0.985, 0.986 and 0.944 for CADM1 (**A**), MAL (**B**) and hsa-miR-124-2 (**C**), respectively. The ΔCq values between singleplex and multiplex qMSP differ, due to differences in set thresholds.

Eventually, the multiplex qMSP was tested on 45 hrHPV-positive cervical scrapings. The normalized ratios of singleplex qMSPs of the cervical scrapings correlated strongly to those of multiplex qMSP for all three markers (Figure 5A-C). Correlation coefficients were as follows: R^2^ = 0.985, R^2^ = 0.986 and R^2^ = 0.944 for CADM1, MAL and hsa-miR-124-2, respectively. Of note, the same Cq ratios between singleplex and multiplex qMSP could be obtained when adjusting the threshold settings for all three markers and ACTB. These results indicate that the developed multiplex qMSP assay shows an equal performance in detection methylation in these samples as the singleplex qMSPs.

## Discussion

Here, we describe the development of a multiplex qMSP analysis for the methylation markers CADM1, MAL and hsa-miR-124-2 and the reference gene ACTB. To obtain a multiplex qMSP, primers were adjusted to acquire identical annealing temperatures to enable similar amplification efficiencies for all targets. Multiplex TaqMan buffer systems were compared and primer concentrations were limited to allow efficient amplification of all targets. Analytical validation using a dilution series of methylated DNA spiked with unmethylated DNA showed an equal performance of the multiplex qMSP compared to the individual singleplex qMSPs. Moreover, the multiplex qMSP was found to be very robust, showing high reproducibility across 10 independent experiments. Further evaluation on cervical scrapings revealed a high correlation between the obtained normalized ratios of the singleplex qMSPs and the multiplex qMSP. Based on our experience obtained by optimizing a multiplex qMSP, a guideline is designed (Figure 1), describing the consecutive experimental steps for multiplex qMSP set up.

Major advantages of multiplexing are less hands-on time and less amounts of DNA required to determine the methylation status of multiple methylation markers as compared to running multiple singleplex analyses. Moreover, freeze-thawing of bisulfite modified DNA, which is inherent to repetitive singleplex analysis may degrade the single-stranded DNA, resulting in increased Cq values [24]. Another advantage is the improved data quality, because the methylation marker is normalized to the reference gene present in the same reaction, thereby reducing the potential negative impact of for example pipetting errors when the reference gene is tested in a separate reaction. These features of multiplex qMSP are extremely beneficial for molecular diagnostics.

Both the EpiTect Methylight and QuantiTect Multiplex Mix buffer systems could be used for multiplex qMSP. Other TaqMan buffer systems, such as the Genotyping Master Mix and the iQ Multiplex Powermix are less favorable for multiplex analysis, due to high Cq values/low ΔRn or low linear phase amplifications. With this optimized multiplex qMSP, the normalized ratios obtained from cervical scrapings correlated strongly with those of the individual singleplex assays.

The development of a multiplex qMSP has been described before in a study on colorectal cancer [25]. In this study the dyes, FAM, Hex CY5 and Texas Red were used, the latter of which prohibits the use of ROX as a passive reference to normalize for non-PCR-related fluctuations in fluorescence signal. Moreover, it is unknown whether the relatively high concentration of ACTB primers used, affected amplification of the other targets, as no primer limiting assays were described. Others have proposed the combination of a circularizing oligonucleotide probe, the so-called target-selection-padlock probes, with microarray technology as a high-throughput approach for DNA methylation detection [26]. However, with this technique hundreds of genes are analyzed, while only several methylation markers are needed for diagnostic use. Another promising multiplex technique is the recently described quantitative allele-specific real-time target and signal amplification (QuARTS), which showed to be highly sensitive [27]. Future studies will show its potential value in a diagnostic setting.

The analytical performance of the multiplex qMSP could potentially be further improved by the incorporation of Locked Nucleic Acids (LNAs) in primers and/or hydrolysis probes [28], as has been described for singleplex MGMT [29] and CADM1 [30] qMSPs. In analogy to its use for improved DNA mutation analysis (as was reviewed by [31]), peptide nucleic acid (PNA) hybridization probes, in which the sugar-phosphate backbone of DNA is replaced by a pseudopeptide and very stable heteroduplexes can be formed. This would prevent amplification of unmethylated DNA, thereby enhancing the analytical performance of qMSP. The impact of such modification on the clinical performance of methylation analysis warrants further evaluation.

The multiplex qMSP described here was performed on an ABI7500 Fast Real-Time PCR System, which supports multiplexing of five targets, when ROX is not present in the buffer system. In principle, even more methylation markers can be incorporated into the multiplex qMSP, when other devices will be used, such as the ViiA™ 7 Real-Time PCR System (Applied Biosystems) or the LightCycler 480 (Roche), which support multiplexing of six targets. We were however unable to develop a multiplex qMSP on the ABI7900HT Fast Real-Time PCR System, which may be related to the differences in detection systems.

The CADM1, MAL, hsa-miR-124-2 multiplex methylation assay described here may serve as a triage test for hrHPV-positive women in population-based screening. We recently demonstrated that combined CADM1 and MAL promoter methylation analysis on physician-taken cervical scrapings of hrHPV-positive women was at least equally discriminatory for high-grade CIN as cytology or cytology combined with HPV16/18 genotyping [14]. Futhermore, these methylation markers are applicable to self-collected cervico-vaginal lavage specimens for colposcopy triage of hrHPV-positive women (Hesselink et. al, unpublished observations) and are currently tested prospectively [32].

## Conclusion

In summary, multiplex qMSP is a high-throughput, quantitative assay to analyze multiple methylation markers in a single reaction. It showed to be equally specific, sensitive and reproducible as its singleplex versions. Due to the material saving capacity, the multiplex qMSP may allow analysis of samples with limited methylated DNA content, such as bodily fluids, like urine, blood or sputum. Multiplex qMSP can be applied to cervical scrapes and self-collected specimens and used as a triage tool for detection of high-grade cervical lesions in hrHPV-positive women.

## Competing interests

Dr. R.D.M. Steenbergen, Dr. D.A.M. Heideman, Prof. dr. P.J.F. Snijders and Prof. dr. C.J.L.M. Meijer have relationships with Self-screen BV, The Netherlands.

## Authors’ contributions

SS developed and analytically validated the multiplex qMSP. LDS and ATH evaluated the multiplex qMSP on cervical scrapings. RDMS and PJFS designed the study and participated in data analysis and interpretation. SS drafted the original manuscript. RDMS, DAMH, PJFS and CJLMM critically revised the manuscript. All authors have read and approved of the final manuscript.

## Pre-publication history

The pre-publication history for this paper can be accessed here:

http://www.biomedcentral.com/1471-2407/12/551/prepub

## Supplementary Material

Additional file 1 **Reproducibility of multiplex qMSP.** Serial dilutions of methylated DNA (SiHa) spiked with unmethylated DNA (EK) in order to obtain a serial dilution of 50 ng to 0.25 ng methylated DNA in a total of 50 ng of DNA, showed high reproducibility when testing the multiplex qMSP in 10-fold. Click here for file
