# Supplementary material for: Development of a multiplex methylation-specific PCR as candidate triage test for women with an HPV-positive cervical scrape
Source: BMC Cancer. 2012 Nov 23;12:551. doi: 10.1186/1471-2407-12-551 (PMC3517769; doi:10.1186/1471-2407-12-551)
Supplement: Additional file 1 — Reproducibility of multiplex qMSP. Serial dilutions of methylated DNA (SiHa) spiked with unmethylated DNA (EK) in order to obtain a serial dilution of 50 ng to 0.25 ng methylated DNA in a total of 50 ng of DNA, showed high reproducibility when testing the multiplex qMSP in 10-fold. [file 1471-2407-12-551-S1.pdf]

Additional file 1

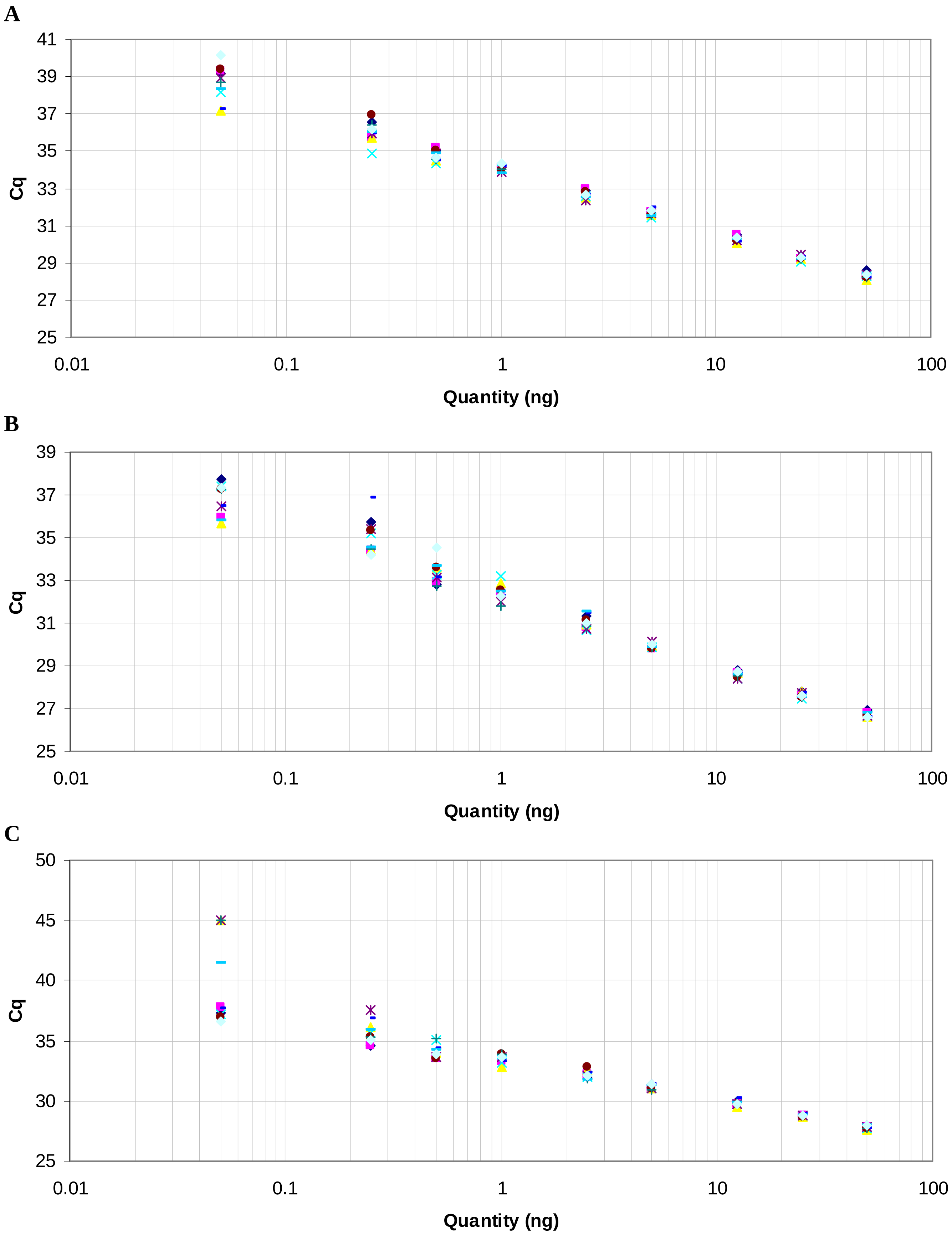

Additional file 1. Reproducibility of multiplex qMSP. Serial dilutions of methylated DNA (SiHa) spiked with unmethylated DNA (EK) in order to obtain a serial dilution of 50 ng to 0.25 ng methylated DNA in a total of 50 ng of DNA, showed high reproducibility when testing the multiplex qMSP in 10-fold.
